# Area Socioeconomic Status, Vaccination Access, and Female Human Papillomavirus Vaccination

**DOI:** 10.1001/jamanetworkopen.2025.0747

**Published:** 2025-03-13

**Authors:** Emiko Oka, Megumi Okada, Yoshiko Ikuno, Kokoro Amano, Sakiko Shioya, Migiri Kawabata, Rie Sakurai, Miki Konishi, Tomoki Nakaya, Kota Katanoda, Yutaka Ueda, Yuri Ito

**Affiliations:** 1 Department of Medical Statistics, Research and Development Center, Osaka Medical and Pharmaceutical University, Osaka, Japan; 2 Department of Obstetrics and Gynecology, Osaka University, Osaka, Japan; 3 Osaka City Public Health Office, Osaka, Japan; 4 Department of Health Promotion, Public Health Bureau, Osaka City, Osaka, Japan; 5 Osaka City Hirano Ward Health and Welfare Center, Osaka, Japan; 6 Osaka City Abeno Ward Health and Welfare Center, Osaka, Japan; 7 Graduate School of Environmental Studies, Tohoku University, Miyagi, Japan; 8 Graduate School of Science, Tohoku University, Miyagi, Japan; 9 Division of Population Data Science, National Cancer Center Institute for Cancer Control, Tokyo, Japan

## Abstract

**Importance:**

Although evaluation of geographic area inequities in vaccination is crucial to identify areas that need community-based interventions, knowledge of disparities in human papillomavirus (HPV) vaccination uptake remains limited in Japan.

**Objective:**

To investigate the association of female cumulative HPV vaccination uptake with neighborhood-based socioeconomic status and access indicators in Osaka City, Japan.

**Design, Setting, and Participants:**

This population-based, cross-sectional study included HPV vaccination data for fiscal year (April 1 to March 31) 2013 to fiscal year 2022 provided by Osaka City. The study population comprised the total number of girls eligible for HPV vaccination born between fiscal years 1997 and 2010.

**Exposure:**

The area deprivation index (ADI) was used as a neighborhood-based socioeconomic status indicator, and the number of medical facilities providing HPV vaccination within a 500-m range of a representative point in each geographic area was used as an access indicator.

**Main Outcomes and Measures:**

The primary outcome was cumulative uptake, defined as the cumulative number of girls receiving at least 1 dose and those completing all doses (3 doses until March 2023), by neighborhood ADI and access indicators. A Poisson regression model with robust variance was applied to assess the association of neighborhood-level indicators with cumulative HPV vaccination uptake.

**Results:**

In Osaka City, 185 373 girls (median [IQR] age at vaccination, 16 [14-19] years) were eligible for HPV vaccination, of whom 18 688 (10.1%) received at least 1 dose of HPV vaccine. Compared with girls living in areas with the most deprivation (2539 of 28 078 [9.0%]), those living in areas with the least deprivation (4889 of 42 170 girls [11.6%]) had a greater cumulative HPV vaccination uptake (prevalence ratio [PR], 1.25; 95% CI, 1.16-1.34). In addition, compared with girls living in areas with low medical facility access (5128 of 55 055 [9.3%]), those residing in high-access areas (5862 of 54 740 [10.7%]) had a greater cumulative vaccination uptake (PR, 1.09; 95% CI, 1.03-1.16). Cumulative HPV vaccination was significantly associated with ADI in routine vaccination (least vs most deprivation: PR, 1.46; 95% CI, 1.33-1.61) but not in catch-up vaccination (least vs most deprivation: PR, 1.01; 95% CI, 0.92-1.11).

**Conclusions and Relevance:**

In this cross-sectional study of area deprivation, vaccination access, and HPV vaccination status, higher socioeconomic status and higher medical facility access were associated with higher cumulative HPV vaccination uptake. These findings suggest that further strategies, including a socioecologic approach, are needed to increase HPV vaccination and reduce disparities in uptake.

## Key Points

**Question:**

What is the association of cumulative human papillomavirus (HPV) vaccination uptake with neighborhood socioeconomic and vaccination access indicators?

**Findings:**

In this cross-sectional study of 185 373 girls eligible for HPV vaccination in Osaka City, Japan, 18 688 received at least 1 dose of the vaccine. Neighborhoods with higher socioeconomic status and higher access to HPV vaccines were associated with significantly higher cumulative HPV vaccination uptake.

**Meaning:**

These findings suggest the need for further strategies, including a socioecologic approach, to reduce inequities in HPV vaccination uptake.

## Teaser

This cross-sectional study investigates the association of human papillomavirus (HPV) vaccination uptake among girls and neighborhood socioeconomic status and access to vaccines in Osaka City, Japan.

## Introduction

Cervical cancer is the fourth most common cancer in women,^1^ and more than 95% of cases have been associated with persistent human papillomavirus (HPV) infection.^2^ Human papillomavirus vaccines are highly effective at preventing infection and subsequent HPV-related diseases.^3^ In 2020, the World Health Organization announced its goal of maintaining an HPV incidence rate of 4 per 100 000 women via 3 strategies, one of which was for 90% of girls to be vaccinated against HPV by age 15 years.^4^ However, HPV vaccination uptake in Japan is the lowest among high-income countries.^5^

Japan has a unique situation regarding HPV vaccination. Historically, vaccination was subsidized for girls in 7th to 10th grade (corresponding to the population born in fiscal years [April 1 to March 31 of the following year] 1994-1997 and fiscal year 2010) as an emergency promotion project^6^ from November 26, 2010, to March 31, 2013. In April 2013, the HPV vaccine was introduced into the national routine vaccination program for girls in 6th to 10th grade (corresponding to the population born in fiscal years 1997-2001 and fiscal year 2013).^7^ Many eligible girls in Japan were vaccinated during this period, and uptake of at least 1 dose temporarily reached 70% to 80%.^6^ However, at this time, the Japanese media began reporting diverse postvaccination symptoms experienced by some girls. In response to these reports, the Ministry of Health, Labour and Welfare suspended its proactive recommendation for HPV vaccination from June 14, 2013, until March 31, 2022.^8,9^ Consequently, uptake among girls who became eligible after the suspension was less than 1%,^10^ and the prevalence of HPV in unvaccinated cohorts was higher than in vaccinated cohorts.^11^ In April 2022, the Ministry of Health, Labour and Welfare announced resumption of its recommendation and offered free catch-up HPV vaccination to individuals born between fiscal years 1997 and 2007 who were not vaccinated during the suspension, even after the period of routine vaccination uptake.^12^ Nonetheless, HPV vaccination coverage in Japan has not recovered.^13^ In this unique Japanese situation, where vaccination coverage varies greatly according to birth year, it is difficult to evaluate HPV vaccination status and trends from year to year. Cumulative HPV vaccination uptake, the proportion of people who have actually been vaccinated against HPV among those who have ever been eligible, could enable us to evaluate HPV vaccination status and its trends each year.

Community-based interventions, including client reminder and recall systems, are needed to increase vaccination uptake.^14,15^ Identification of areas of low uptake may allow such interventions to be targeted to where they will be most effective.^14^ A previous study reported uptake to be higher in affluent areas,^16^ while another found it to be higher among girls living in urban areas, possibly due to poor health care access in rural areas.^17^ Although investigation into the association between uptake and neighborhood-based indicators in Japan is necessary, studies of area-based differences in HPV vaccination are scarce.

Osaka City is a metropolitan area located in western Japan.^18^ Osaka Prefecture has the second highest number of universities and companies in Japan after Tokyo.^18,19^ In Osaka City, data on all individuals vaccinated against HPV since 2013, when the routine vaccination program was introduced, are stored, facilitating detailed analysis. Thus, we used these data to calculate cumulative HPV vaccination uptake in Osaka City and investigate the association between cumulative uptake and neighborhood-based socioeconomic and access indicators.

## Methods

This cross-sectional study used local government administrative population data. It was approved by the Osaka Medical and Pharmaceutical University Hospital Ethics Committee. Exemption of written informed consent was granted by the Ethics Committee because of the anonymity of the data. This study followed the Strengthening the Reporting of Observational Studies in Epidemiology (STROBE) reporting guideline.

We included girls born between fiscal years 1997 and 2010 who received HPV vaccination in Osaka City from fiscal years 2013 through 2022. They were eligible for the national routine HPV vaccination program and the catch-up HPV vaccination program even after the period of routine vaccination. Girls born between fiscal years 1994 and 1996 are eligible for subsidized HPV vaccination, and many were reported as already vaccinated^10^ but were excluded because no individual data, including residential area, existed and they were not eligible for the catch-up HPV vaccination program.

On the basis of a previous report,^20^ we categorized girls’ birth fiscal year as the vaccination generation (born between fiscal years 1997 and 1999), vaccine suspension generation (born between fiscal years 2000 and 2005), and reintroduction generation (born after fiscal year 2006) and their vaccination periods as the suspension period (vaccinated between fiscal years 2013 and 2021) and resumption period (vaccinated in fiscal year 2022). A chart showing the correspondence of calendar year with fiscal year in Japan is provided in eTable 1 in Supplement 1. The school year corresponds to the fiscal year, and girls eligible for HPV vaccination were determined by birth fiscal year.

### Data Sources

#### HPV Vaccination Data

We used individual HPV vaccination data from fiscal years 2013-2022 provided by Osaka City, including routine and catch-up vaccination data. The data also included birth year, vaccination year, number of HPV vaccinations, and residential area at time of vaccination. Vaccination data from November 26, 2010, to March 31, 2013, did not include individual information on residential area. Thus, the tabulated data on the number of girls vaccinated for this period were used to assess the current HPV vaccination status in the whole of Osaka City in addition to the data between fiscal years 2013 and 2022 and were not used in the analyses of the association between neighborhood-level indicators and cumulative vaccination uptake. The number of vaccinated girls by vaccination fiscal year and by residential area at the time of vaccination was defined as the cumulative number of girls vaccinated against HPV.

#### Data on Population Eligible for HPV Vaccination

Population data by 1-year age groups and small areas based on the Basic Resident Registration System from the Osaka City open data portal website were used to calculate the population of the age groups eligible for HPV vaccination.^21^ The population eligible for HPV vaccination was defined as the total female population born after fiscal year 1997 and eligible for free HPV vaccination (eFigure 1 in Supplement 1).

### Variables

#### Cumulative HPV Vaccination Uptake

Cumulative HPV vaccination uptake for at least 1 dose and for completed doses (ie, 3 doses until March 2023) was equal to the cumulative number of girls vaccinated divided by the total female population eligible for HPV vaccination. We used the cumulative number of girls vaccinated against HPV since fiscal year 2010 for the whole of Osaka City and the cumulative number of girls vaccinated against HPV since fiscal year 2013 for each small area because individual data with residential area at time of vaccination were only available from fiscal year 2013 onward.

To compare the status of HPV vaccination in Osaka City with the whole of Japan, we collected the number of vaccinations throughout Japan.^10,22,23^ Cumulative uptake for at least 1 dose for the whole of Japan was calculated.

#### Neighborhood-Based Indicators

We used 2 variables for neighborhood-based indicators: a socioeconomic indicator and an access indicator. The area deprivation index (ADI) is a Japanese ecologic socioeconomic deprivation index.^24^ The ADI was initially developed in the UK, was subsequently adopted by other countries,^25,26^ and has been widely used in Japan for the evaluation of socioeconomic disparities in a variety of health-related outcomes.^27,28,29,30^ In this study, we calculated the ADI by *Cho-Aza*, the country’s smallest administrative unit, with an average of 752 households per unit. In 2023, there were 1902 *Cho-Aza* units in Osaka City. We categorized deprivation level into quintile groups weighted by the number of households (from least deprivation in the first quintile to most deprivation in the fifth quintile).

We also counted the number of medical facilities providing HPV vaccination within 500 m of a representative point in each geographic unit as an index of access to HPV vaccination and categorized this into tertile groups weighted by the number of households (0-6 facilities in the first tertile [lowest access], 7-10 facilities in the second, 11-26 facilities in the third [highest access]).

### Statistical Analysis

For the total, routine, and catch-up vaccination programs, we calculated cumulative HPV vaccination uptake with at least 1 dose and with completed doses by vaccination year in Osaka City. Cumulative uptake by ADI and access indicator was also calculated. The sum of target population and cumulative HPV vaccination in ADI can be less than the sum in the access indicator because ADI cannot be calculated in areas with fewer than 50 households. The Jonckheere-Terpstra test was used to assess trends in cumulative HPV vaccination uptake by vaccination year and each neighborhood-based indicator, with *P* < .05 indicating significant differences.

A Poisson regression model with a robust error variance was applied to assess the association of ADI and access to medical facilities providing HPV vaccination with the cumulative proportion of HPV vaccinations given. Prevalence ratios (PRs) are presented, with areas with the most deprivation and lowest access as the reference. Multivariable analysis was adjusted for ADI, access indicators, generation of birth fiscal year, and vaccination period. We also assessed the interaction between ADI and access to evaluate access-based differences in ADI and cumulative HPV vaccination uptake. All statistical analyses were performed using Stata, version 18.0 (StataCorp LLC). When no overlap in the 95% CI was observed, the difference was regarded as statistically significant.

## Results

### HPV Vaccination Uptake in the Whole of Osaka City

**Figure 1.**
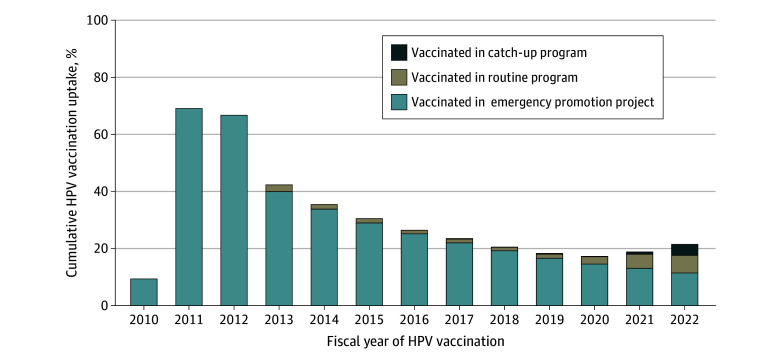
Cumulative Human Papillomavirus (HPV) Vaccination Uptake of at Least 1 Dose in Osaka City, Japan

**Figure 2.**
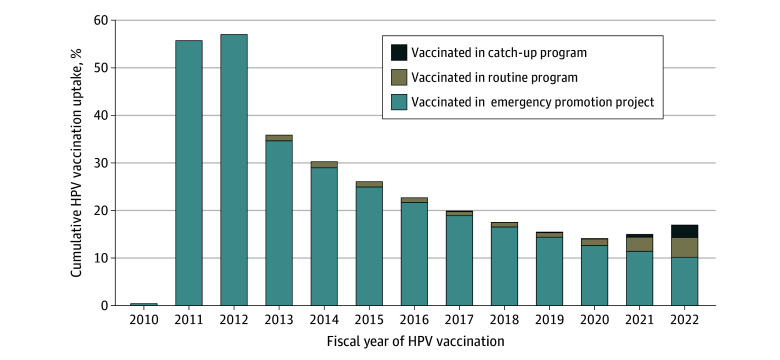
Cumulative Human Papillomavirus (HPV) Vaccination Uptake (Completed Dose) in Osaka City, Japan Completed dose indicates 3 doses of vaccine until March 2023.

In Osaka City, of a total of 185 373 eligible girls (median [IQR] age at vaccination, 16 [14-19] years), 18 688 (10.1%) were vaccinated between fiscal years 2013 and 2022. Including tabulated HPV vaccination data from November 2010 to March 2013, the cumulative uptake of at least 1 dose of the HPV vaccine in the whole of Osaka City rose to 69.5% (14 397 of 20 712 girls) in fiscal year 2011 but dropped to 17.4% (25 281 of 145 427 girls) in fiscal year 2020 during the vaccination recommendation suspension and rose to 21.6% (40 126 of 185 373 girls) in fiscal year 2022 (Figure 1; eTable 2 in Supplement 1). Cumulative completed dose HPV vaccination uptake in 2022 was 16.8% (31 178 of 185 373 girls) (Figure 2). In addition, 21 438 of 40 216 girls (53.3%) vaccinated before 2022 had received the vaccine between 2010 and 2012 before the suspension. Cumulative HPV vaccination uptake with at least 1 dose was higher in the whole of Japan in 2022 (28.1%) than in Osaka City (21.6%) (eTables 2 and 3A, and eFigure 2 in Supplement 1). Cumulative HPV vaccination uptake with at least 1 dose in the whole of Japan in 2022 was higher among girls born between fiscal years 1994 and 2010 (35.1%) than between fiscal years 1997 and 2010 (28.1%) (eTable 3A and B in Supplement 1).

### Neighborhood-Based Disparities in Cumulative HPV Vaccination Uptake

Among the 18 688 girls vaccinated in Osaka City, those living in areas with the least deprivation and highest access were more likely to receive routine HPV vaccination (66.6% and 62.6%, respectively), were born in the vaccine suspension generation (53.2% and 53.6%, respectively), and received HPV vaccination in the suspension period (52.6% and 50.7%, respectively) (Table 1). Cumulative HPV vaccination uptake in areas with the least deprivation and highest access was higher than areas with the most deprivation and lowest access (least vs most deprivation, 4889 of 42 170 girls [11.6%] vs 2539 of 28 078 girls [9.0%]; lowest vs highest access, 5128 of 55 055 girls [9.3%] vs 5862 of 54 740 girls [10.7%]) (Table 2). We compared cumulative HPV vaccination in fiscal year 2013, when the national vaccination program started; in fiscal year 2020, when uptake was lowest; and in fiscal year 2022, when vaccination increased again. The difference in cumulative vaccination between the areas with the least and most deprivation gradually widened throughout these periods (0.7% in fiscal year 2013, 1.5% in fiscal year 2020, and 2.6% in fiscal year 2022) (eTable 4 in Supplement 1). The pattern of completed doses was similar to that of at least 1 dose (eTable 5 in Supplement 1).

**Table 1.** Characteristics of Girls Vaccinated for HPV by Neighborhood-Based Indicators (n = 18 688), Osaka City, Japan

Characteristic	Girls, No. (%)							
	Area deprivation index quintile^a^					Access to HPV vaccination tertile^b^		
	1 (least deprivation)	2	3	4	5 (most deprivation)	1 (lowest)	2	3 (highest)
No. of girls (%)	4889 (26.2)	4041 (21.6)	3696 (19.8)	3519 (18.8)	2539 (13.6)	5128 (27.4)	7698 (41.2)	5862 (31.4)
Routine vaccination	3256 (66.6)	2456 (60.8)	2257 (61.1)	2057 (58.5)	1420 (55.9)	3069 (59.8)	4709 (61.2)	3670 (62.6)
Catch-up vaccination	1633 (33.4)	1585 (39.2)	1439 (38.9)	1462 (41.5)	1119 (44.1)	2059 (40.2)	2989 (38.8)	2192 (37.4)
Generation of birth fiscal year^c^								
Vaccination	427 (8.7)	450 (11.1)	467 (12.6)	522 (14.8)	430 (16.9)	720 (14)	946 (12.3)	631 (10.8)
Vaccine suspension	2603 (53.2)	2211 (54.7)	1930 (52.2)	1871 (53.2)	1290 (50.8)	2642 (51.5)	4123 (53.6)	3141 (53.6)
Reintroduction	1859 (38)	1380 (34.1)	1299 (35.1)	1126 (32)	819 (32.3)	1766 (34.4)	2629 (34.2)	2090 (35.7)
Vaccination period^d^								
Suspension	2570 (52.6)	1965 (48.6)	1829 (49.5)	1684 (47.9)	1148 (45.2)	2399 (46.8)	3826 (49.7)	2971 (50.7)
Resumption	2319 (47.4)	2076 (51.4)	1867 (50.5)	1835 (52.1)	1391 (54.8)	2729 (53.2)	3872 (50.3)	2891 (49.3)

Abbreviation: HPV, human papillomavirus.

^a^ The total sum of the target population and cumulative HPV vaccination in the area deprivation index is less than that in the access indicator because the area deprivation index cannot be calculated in areas with fewer than 50 households.

^b^ Number of facilities are as follows: tertile 1, 0 to 5 facilities; tertile 2, 6 to 10 facilities; and tertile 3, 11 to 26 facilities.

^c^ Definitions of generations are as follows: vaccination, girls born in fiscal years 1997-1999; vaccine suspension, girls born in fiscal years 2000-2005; and reintroduction, girls born in fiscal years 2006-2010.

^d^ Definitions of vaccination period are as follows: suspension, fiscal years 2013-2021, and resumption, fiscal year 2022.

**Table 2.** Cumulative HPV Vaccination Coverage of at Least 1 Dose by Neighborhood-Based Indicators in 2022

Exposure	Target population, No.^a^	Cumulative HPV vaccination, No. (%)^a^	*P* value^b^
**Routine plus catch-up vaccination**			
Area deprivation index quintile			
5 (most deprivation)	28 078	2539 (9.0)	.01
4	36 846	3519 (9.6)	
3	38 631	3696 (9.6)	
2	39 495	4041 (10.2)	
1 (least deprivation)	42 170	4889 (11.6)	
Access to HPV vaccination tertile^c^			
1 (lowest)	55 055	5128 (9.3)	.11
2	75 578	7698 (10.2)	
3 (highest)	54 740	5862 (10.7)	
**Routine vaccination**			
Area deprivation index quintile			
5 (most deprivation)	28 078	1420 (5.1)	.01
4	36 846	2057 (5.6)	
3	38 631	2257 (5.8)	
2	39 495	2456 (6.2)	
1 (least deprivation)	42 170	3256 (7.7)	
Access to HPV vaccination tertile^c^			
1 (lowest)	55 055	3069 (5.6)	.11
2	75 578	4709 (6.2)	
3 (highest)	54 740	3670 (6.7)	
**Completed dose**			
Area deprivation index quintile			
5 (most deprivation)	28 078	1119 (4.0)	.62
4	36 846	1462 (4.0)	
3	38 631	1439 (3.7)	
2	39 495	1585 (4.0)	
1 (least deprivation)	42 170	1633 (3.9)	
Access to HPV vaccination tertile^c^			
1 (lowest)	55 055	2059 (3.7)	.12
2	75 578	2989 (4.0)	
3 (highest)	54 740	2192 (4.0)	

Abbreviation: HPV, human papillomavirus.

^a^ The total sum of target population and cumulative HPV vaccination in the area deprivation index is less than that in the access indicator because the area deprivation index cannot be calculated in areas with fewer than 50 households.

^b^ The Jonckheere-Terpstra test was performed to identify trends in cumulative HPV vaccination coverage.

^c^ Number of facilities are as follows: tertile 1, 0 to 5 facilities; tertile 2, 6 to 10 facilities; and tertile 3, 11 to 26 facilities.

For the routine and catch-up vaccination programs, HPV vaccination uptake in the areas of least deprivation and highest access was significantly higher than in the areas with the most deprivation and lowest access areas (least vs most deprivation: PR, 1.25 [95% CI, 1.16-1.34]; highest vs lowest access: PR, 1.09 [95% CI, 1.03-1.16]) (Table 3). The association between ADI and cumulative vaccination uptake was stronger than that between access and cumulative vaccination uptake. In stratified analysis, cumulative HPV vaccination was significantly associated with ADI in routine vaccination (least vs most deprivation: PR, 1.46; 95% CI, 1.33-1.61) but not in catch-up vaccination (least vs most deprivation: PR, 1.01; 95% CI, 0.92-1.11). The pattern of completed doses was similar to that for at least 1 dose (eTable 6 in Supplement 1). The interaction between ADI and access was 1.3 times higher for cumulative HPV vaccination uptake in the areas of least deprivation and highest access than in the areas of most deprivation and lowest access (pairwise correlation coefficient, −0.3) (eTables 7 and 8 in Supplement 1).

**Table 3.** Association Between Neighborhood-Based Indicators and Cumulative HPV Vaccination Coverage of at Least 1 Dose in 2022

Exposure	PR (95% CI)	
	Univariable analysis	Multivariable analysis^a^
**Routine plus catch-up vaccination**		
Area deprivation index quintile		
5 (most deprivation)	1 [Reference]	1 [Reference]
4	1.06 (0.98-1.13)	1.04 (0.97-1.11)
3	1.06 (0.98-1.14)	1.03 (0.96-1.11)
2	1.13 (1.05-1.22)	1.10 (1.02-1.19)
1 (least deprivation)	1.28 (1.19-1.38)	1.25 (1.16-1.34)
Access to HPV vaccination tertile^b^		
1 (lowest)	1 [Reference]	1 [Reference]
2	1.09 (1.03-1.16)	1.08 (1.02-1.14)
3 (highest)	1.15 (1.08-1.22)	1.09 (1.03-1.16)
**Routine vaccination**		
Area deprivation index quintile		
5 (most deprivation)	1 [Reference]	1 [Reference]
4	1.10 (0.99-1.22)	1.10 (1.00-1.20)
3	1.16 (1.04-1.28)	1.13 (1.03-1.24)
2	1.23 (1.11-1.36)	1.23 (1.12-1.35)
1 (least deprivation)	1.53 (1.38-1.69)	1.46 (1.33-1.61)
Access to HPV vaccination tertile^b^		
1 (lowest)	1 [Reference]	1 [Reference]
2	1.12 (1.03-1.21)	1.08 (1.01-1.16)
3 (highest)	1.20 (1.11-1.31)	1.10 (1.02-1.18)
**Catch-up vaccination**		
Area deprivation index quintile		
5 (most deprivation)	1 [Reference]	1 [Reference]
4	1.00 (0.89-1.11)	0.98 (0.90-1.08)
3	0.93 (0.84-1.04)	0.94 (0.85-1.03)
2	1.01 (0.90-1.13)	0.99 (0.90-1.09)
1 (least deprivation)	0.97 (0.87-1.08)	1.01 (0.92-1.11)
Access to HPV vaccination tertile^b^		
1 (lowest)	1 [Reference]	1 [Reference]
2	1.06 (0.98-1.14)	1.08 (1.00-1.15)
3 (highest)	1.07 (0.99-1.16)	1.11 (1.03-1.20)

Abbreviations: HPV, human papillomavirus; PR, prevalence ratio.

^a^ The multivariable analysis was adjusted as follows: area deprivation index, access to medical facilities providing HPV vaccination, generation of birth fiscal year, and vaccination period.

^b^ Number of facilities are as follows: tertile 1, 0 to 5 facilities; tertile 2, 6 to 10 facilities; and tertile 3, 11 to 26 facilities.

## Discussion

This cross-sectional study shows that cumulative HPV vaccination uptake for at least 1 dose in 2022 was 21.6% in Osaka City, Japan. It dropped to 17.4% in 2020 and recovered afterward. This study is the first to our knowledge of disparities in HPV vaccination uptake using neighborhood-based indicators in Japan. The findings show that there were inequities in cumulative uptake according to neighborhood-based socioeconomic and access indicators. Cumulative uptake in areas with the most deprivation and lowest access was lower than in areas of least deprivation and highest access. Disparities in routine HPV vaccination were greater than in catch-up HPV vaccination.

Cumulative HPV vaccination uptake in Osaka City plunged in 2013 and began to rise in 2021. In response to accumulated evidence on HPV vaccination,^31,32^ some municipalities in Japan started individual notification of HPV vaccination in October 2020, and in November 2021, the Ministry of Health, Labour and Welfare resumed its recommendation for HPV vaccination, starting the following year. The improvement in cumulative uptake in Osaka may largely be the result of efforts to promote HPV vaccination by municipalities and the government’s decision to resume its recommendation.

Cumulative uptake in Osaka City was lower than in the whole of Japan, though the trend in uptake was similar. A major reason for this finding may be population shifts. Osaka City is a metropolitan city to which many people move after graduating from high school. Our targeted population for catch-up HPV vaccination included females who had already been vaccinated with more than 1 dose in their childhood hometown, which was possibly the case in urban Osaka City and may have influenced the cumulative HPV vaccination uptake.

Our finding indicating higher cumulative vaccination uptake in more affluent areas was similar to previous studies.^16,33,34^ One reason for this disparity may be lack of access. In our study, the areas with the most deprivation had lower medical facility delivery of HPV vaccination (pairwise correlation coefficient, −0.3), and the prevalence ratio of ADI for cumulative uptake decreased after adjusting for access. Another reason may be that people living in areas of more deprivation may have poor health behaviors.^28,35^ In such areas, promoting HPV vaccination in schools has been reported to be effective.^36^

Our findings suggest that improving access, including increasing the number of facilities offering HPV vaccination, may play an important role in increasing uptake. However, no statistically significant association between vaccination uptake and access was observed in areas with the most deprivation (eTable 8 in Supplement 1), suggesting that other factors may be involved in access besides the number of facilities. Some reports have highlighted the challenges faced by residents of areas with low uptake, such as travel distance and transportation problems.^17,37,38^ These factors need to be explored further.

Neighborhood-level disparities in HPV vaccination suggest an urgent need to strengthen approaches to areas with low uptake. For example, in community-level interventions, school-based HPV vaccination is one of the most influential strategies in increasing uptake and reducing inequities.^39,40,41^ Clinic-level interventions, such as patient education, may be more effective when combined with other interventions, such as reduction of patient out-of-pocket costs.^14,15,42^ In addition, effective interventions may differ by socioeconomic level or urban/rural status of the area.^36,43^ Our findings may help identify the characteristics of regions requiring intervention to increase HPV vaccination uptake.

### Strengths and Limitations

A strength of our study is the use of population-based data, including residential area and demonstrated neighborhood-based socioeconomic inequities and influence of access to clinics for HPV vaccination in Japan. The results enabled us to identify high-priority regions requiring intervention, including new approaches to reach people who have not been vaccinated (eg, additional vaccination venues) to increase uptake. For our next step in this research, we need to monitor the effectiveness of vaccination. In Japan, we do not have a comprehensive system that links vaccinations, screenings, and results. It is time to establish such a system to manage screening programs and the results in order to create an effective total prevention program for cervical cancer.

Our study also has several limitations. First, we did not control for potential confounding variables, including health literacy and attitudes toward HPV vaccination, because we did not have access to such information. However, ADI could be an alternative indicator of health literacy and family income levels. Urbanization was not controlled to prevent multicollinearity. Differences in specialties were not evaluated because information on medical facilities delivering HPV vaccination in Osaka City did not include specialties. Further analysis using data that include these specialties may be helpful to assess the uptake of HPV vaccination by specialty.

Second, we excluded girls born between fiscal years 1994 and 1996 from the analyses, although a substantial number in this age group were vaccinated before the government suspended its recommendation, which may have led to an underestimation of the cumulative uptake in Osaka City. In fact, the cumulative uptake in the whole of Japan, including those born after fiscal year 1994, was 35.1% in 2022 (vs 28.1% for those born after fiscal year 1997) (eTable 3A and B in Supplement 1). Nevertheless, our aim was to evaluate the association between neighborhood-level indicators and HPV vaccination status, so we excluded girls born between fiscal years 1994 and 1996 because there were no individual data on residential area.

Third, our population-based dataset had a limited geographic range, with data only for Osaka City, not for the whole of Japan. Human papillomavirus vaccination data from all of Japan were needed to determine whether our results could be generalized to the whole country, but data for small administrative units were not available. We hope that detailed HPV vaccination data will be gathered and made accessible in the near future.

## Conclusions

In this population-based, cross-sectional study of area deprivation and HPV vaccination access and status, cumulative HPV vaccination uptake was higher in areas of least deprivation and highest access to HPV vaccination than in areas of the most deprivation and lowest access. Our findings suggest that girls living in low-income and low–health care access areas need more support to become vaccinated for HPV.

## Notes

Supplement 1. **eFigure 1.** Age by Birth Fiscal Year **eFigure 2.** Cumulative HPV Vaccination Uptake in Japan (at Least 1 Dose) **eTable 1.** Correspondence Between Calendar Year and Fiscal Year **eTable 2.** Trend of Cumulative HPV Vaccination Coverage in Osaka City **eTable 3A.** Trend of Cumulative HPV Vaccination Coverage With First Dose in Japan (Including Those Born After Fiscal Year 1997) **eTable 3B.** Trend of Cumulative HPV Vaccination Coverage With First Dose in Japan (Including Those Born After Fiscal Year 1994) **eTable 4.** Change of Cumulative HPV Vaccination by Neighborhood-Level Indicators **eTable 5.** Cumulative Completed Dose HPV Vaccination Coverage by Neighborhood-Based Indicators in 2022 **eTable 6.** Association Between Neighborhood-Based Indicators and Cumulative Completed Dose HPV Vaccination Coverage in 2022 **eTable 7.** Cross-Tabulation of Cumulative at Least 1-Dose HPV Vaccination Coverage in 2022 by ADI and Access **eTable 8.** Interaction Between ADI and Access

Supplement 2.
Data Access Statement
