# Supplementary material for: Area Socioeconomic Status, Vaccination Access, and Female Human Papillomavirus Vaccination
Source: JAMA Netw Open. 2025 Mar 13;8(3):e250747. doi: 10.1001/jamanetworkopen.2025.0747 (PMC11907311; doi:10.1001/jamanetworkopen.2025.0747)
Supplement: Supplement 1. — eFigure 1. Age by Birth Fiscal Year eFigure 2. Cumulative HPV Vaccination Uptake in Japan (at Least 1 Dose) eTable 1. Correspondence Between Calendar Year and Fiscal Year eTable 2. Trend of Cumulative HPV Vaccination Coverage in Osaka City eTable 3A. Trend of Cumulative HPV Vaccination Coverage With First Dose in Japan (Including Those Born After Fiscal Year 1997) eTable 3B. Trend of Cumulative HPV Vaccination Coverage With First Dose in Japan (Including Those Born After Fiscal Year 1994) eTable 4. Change of Cumulative HPV Vaccination by Neighborhood-Level Indicators eTable 5. Cumulative Completed Dose HPV Vaccination Coverage by Neighborhood-Based Indicators in 2022 eTable 6. Association Between Neighborhood-Based Indicators and Cumulative Completed Dose HPV Vaccination Coverage in 2022 eTable 7. Cross-Tabulation of Cumulative at Least 1-Dose HPV Vaccination Coverage in 2022 by ADI and Access eTable 8. Interaction Between ADI and Access [file jamanetwopen-e250747-s001.pdf]

Supplemental Online Content

Oka E, Okada M, Ikuno Y, et al. Area socioeconomic status, vaccination access, and female human papillomavirus vaccination. *JAMA Netw Open*. 2025;8(3):e250747. doi:10.1001/jamanetworkopen.2025.0747

eFigure 1. Age by Birth Fiscal Year
eFigure 2. Cumulative HPV Vaccination Uptake in Japan (at Least 1 Dose)
eTable 1. Correspondence Between Calendar Year and Fiscal Year
eTable 2. Trend of Cumulative HPV Vaccination Coverage in Osaka City
eTable 3A. Trend of Cumulative HPV Vaccination Coverage With First Dose in Japan (Including Those Born After Fiscal Year 1997)
eTable 3B. Trend of Cumulative HPV Vaccination Coverage With First Dose in Japan (Including Those Born After Fiscal Year 1994)
eTable 4. Change of Cumulative HPV Vaccination by Neighborhood-Level Indicators
eTable 5. Cumulative Completed Dose HPV Vaccination Coverage by Neighborhood-Based Indicators in 2022
eTable 6. Association Between Neighborhood-Based Indicators and Cumulative Completed Dose HPV Vaccination Coverage in 2022
eTable 7. Cross-Tabulation of Cumulative at Least 1-Dose HPV Vaccination Coverage in 2022 by ADI and Access
eTable 8. Interaction Between ADI and Access

This supplemental material has been provided by the authors to give readers additional information about their work.

© 2025 Oka E et al. *JAMA Network Open*.

eFigure 1. Age by Birth Fiscal Year

Fiscal year of HPV Vaccination

Birth fiscal year	2010	2011	2012	2013	2014	2015	2016	2017	2018	2019	2020	2021	2022
1994	16	17	18	19	20	21	22	23	24	25	26	27	28
1995	15	16	17	18	19	20	21	22	23	24	25	26	27
1996	14	15	16	17	18	19	20	21	22	23	24	25	26
1997	13	14	15	16	17	18	19	20	21	22	23	24	25
1998	12	13	14	15	16	17	18	19	20	21	22	23	24
1999	11	12	13	14	15	16	17	18	19	20	21	22	23
2000	10	11	12	13	14	15	16	17	18	19	20	21	22
2001	9	10	11	12	13	14	15	16	17	18	19	20	21
2002	8	9	10	11	12	13	14	15	16	17	18	19	20
2003	7	8	9	10	11	12	13	14	15	16	17	18	19
2004	6	7	8	9	10	11	12	13	14	15	16	17	18
2005	5	6	7	8	9	10	11	12	13	14	15	16	17
2006	4	5	6	7	8	9	10	11	12	13	14	15	16
2007	3	4	5	6	7	8	9	10	11	12	13	14	15
2008	2	3	4	5	6	7	8	9	10	11	12	13	14
2009	1	2	3	4	5	6	7	8	9	10	11	12	13
2010	0	1	2	3	4	5	6	7	8	9	10	11	12

Age publicly subsidized for emergency promotion project

Age eligible for routine program

Age eligible for catch-up program

Total age eligible for HPV vaccination

Abbreviations: HPV, Human Papillomavirus

eFigure 2. Cumulative HPV Vaccination Uptake in Japan (at Least One Dose)

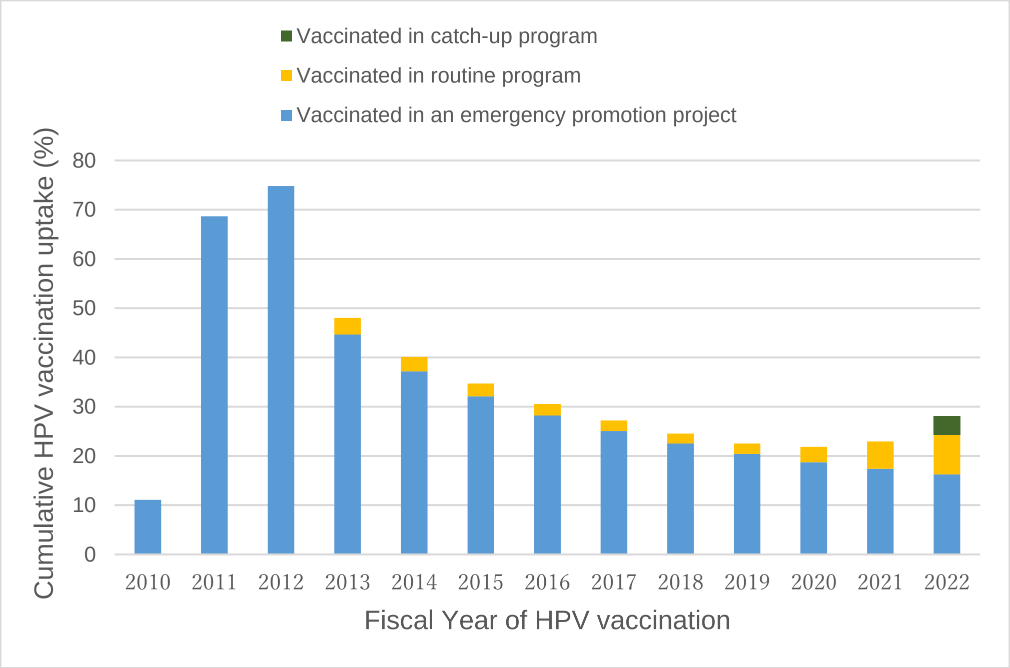

Abbreviations: HPV, Human Papillomavirus

© 2025 Oka E et al. *JAMA Network Open*.

eTable 1. Correspondence Between Calendar Year and Fiscal Year

<table>
<tr><th>Months</th><th>Calendar Year</th><th>Fiscal Year</th></tr>
<tr><td>January</td><td rowspan="12">2020</td><td rowspan="3">2019</td></tr>
<tr><td>February</td></tr>
<tr><td>March</td></tr>
<tr><td>April</td><td rowspan="12">2020</td></tr>
<tr><td>May</td></tr>
<tr><td>June</td></tr>
<tr><td>July</td></tr>
<tr><td>August</td></tr>
<tr><td>September</td></tr>
<tr><td>October</td></tr>
<tr><td>November</td></tr>
<tr><td>December</td></tr>
<tr><td>January</td><td rowspan="3">2021</td></tr>
<tr><td>February</td></tr>
<tr><td>March</td></tr>
</table>

© 2025 Oka E et al. *JAMA Network Open*.

eTable 2. Trend of Cumulative HPV Vaccination Coverage in Osaka City

Year at Vaccination	Target population	Cumulative HPV Vaccination for emergency promotion project, No. (%)	Cumulative HPV Vaccination for routine program, No. (%)	Cumulative HPV Vaccination for catch-up program, No. (%)	Cumulative HPV vaccination coverage (%, an emergency program + routine program + catch-up program)
**At least 1 dose**					
2010	10201	965 (9.5)	0 (0)	0 (0)	9.5
2011	20712	14397 (69.5)	0 (0)	0 (0)	69.5
2012	32033	21438 (66.9)	0 (0)	0 (0)	66.9
2013	52859	21438 (40.6)	936 (1.8)	0 (0)	42.3
2014	63065	21438 (34.0)	1033 (1.6)	0 (0)	35.6
2015	73523	21438 (29.2)	1077 (1.5)	0 (0)	30.6
2016	84626	21438 (25.3)	1120 (1.3)	0 (0)	26.7
2017	96958	21438 (22.1)	1192 (1.2)	1 (0.001)	23.3
2018	111004	21438 (19.3)	1393 (1.3)	11 (0.01)	20.6
2019	128154	21438 (16.7)	1814 (1.4)	22 (0.02)	18.2
2020	145427	21438 (14.7)	3748 (2.6)	95 (0.07)	17.4
2021	162086	21438 (13.2)	7939 (4.9)	1257 (0.8)	18.9
2022	185373	21438 (11.6)	11448 (6.2)	7240 (3.9)	21.6
**Completed dose**					
2010	10201	29 (0.3)	0 (0)	0 (0)	0.3
2011	20712	11552 (55.8)	0 (0)	0 (0)	55.8
2012	32033	18271 (57.0)	0 (0)	0 (0)	57.0
2013	52859	18271 (34.6)	639 (1.2)	0 (0)	35.8
2014	63065	18271 (29.0)	755 (1.2)	0 (0)	30.2
2015	73523	18271 (24.9)	809 (1.1)	0 (0)	26.0
2016	84626	18271 (21.6)	845 (1.0)	0 (0)	22.6
2017	96958	18271 (18.8)	882 (0.9)	2 (0.002)	19.8
2018	111004	18271 (16.5)	988 (0.9)	5 (0.005)	17.4
2019	128154	18271 (14.3)	1214 (0.9)	24 (0.02)	15.2
2020	145427	18271 (12.6)	1943 (1.3)	52 (0.04)	13.9
2021	162086	18271 (11.3)	4844 (3.0)	1022 (0.6)	14.9
2022	185373	18271 (9.9)	7968 (4.3)	4939 (2.7)	16.8

Abbreviations: HPV, Human Papillomavirus

© 2025 Oka E et al. *JAMA Network Open.*

eTable 3A. Trend of Cumulative HPV Vaccination Coverage With First Dose in Japan (Including Those Born After Fiscal Year 1997)

Year at Vaccination	Target population	Number of females vaccinated (routine vaccination)	Cumulative HPV vaccination uptake (%, routine vaccination)	Number of females vaccinated (catch-up vaccination)	Cumulative HPV vaccination uptake (%, catch-up vaccination)	Cumulative HPV vaccination uptake (%, routine + catch-up vaccination)
2010	581612	64513	11.1	0	0	11.1
2011	1161696	797729	68.7	0	0	68.7
2012	1741105	1302711	74.8	0	0	74.8
2013	2918212	1401367	48.0	0	0	48.0
2014	3503065	1405262	40.1	0	0	40.1
2015	4058171	1407973	34.7	0	0	34.7
2016	4615892	1409807	30.5	0	0	30.5
2017	5196361	1413154	27.2	0	0	27.2
2018	5786070	1419964	24.5	0	0	24.5
2019	6379331	1437261	22.5	0	0	22.5
2020	6958608	1520996	21.9	0	0	21.9
2021	7490996	1719470	23.0	0	0	23.0
2022	8007760	1945463	24.3	304737	3.8	28.1

© 2025 Oka E et al. *JAMA Network Open*.

eTable 3B. Trend of Cumulative HPV Vaccination Coverage With First Dose in Japan (Including Those Born After Fiscal Year 1994)

Year at Vaccination	Target population	Number of females vaccinated (routine vaccination)	Cumulative HPV vaccination uptake (%, routine vaccination)	Number of females vaccinated (catch-up vaccination)	Cumulative HPV vaccination uptake (%, catch-up vaccination)	Cumulative HPV vaccination uptake (%, routine + catch-up vaccination)
2010	2344077	378114	16.1	0	0	16.1
2011	2924458	1967323	67.3	0	0	67.3
2012	3499461	2517384	71.9	0	0	71.9
2013	4695770	2616040	55.7	0	0	55.7
2014	5311460	2619919	49.3	0	0	49.3
2015	5879218	2622616	44.6	0	0	44.6
2016	6457434	2624450	40.6	0	0	40.6
2017	7044340	2627797	37.3	0	0	37.3
2018	7644136	2634607	34.5	0	0	34.5
2019	8254099	2651904	32.1	0	0	32.1
2020	8835297	2735639	31.0	0	0	31.0
2021	9364312	2934113	31.3	0	0	31.3
2022	9872321	3160106	32.0	304737	3.1	35.1

© 2025 Oka E et al. *JAMA Network Open*.

eTable 4. Change of Cumulative HPV Vaccination by Neighborhood-Level Indicators

Exposure	Target population in 2013 No.[a]	Cumulative HPV Vaccination in 2013 No. (%)[a]	PR (95%CI)[b]	Target population in 2020 No.[a]	Cumulative HPV Vaccination in 2020 No. (%)[a]	PR (95%CI)[b]	Target population in 2022 No.[a]	Cumulative HPV Vaccination in 2022 No. (%)[a]	PR (95%CI)[b]
**Routine + Catch-up vaccination**									
ADI									
Q5 (Most deprived)	9493	143 (1.5)	Ref.	23046	468 (2)	Ref.	28078	2539 (9)	Ref.
Q4	11017	208 (1.9)	1.06 (0.89-1.26)	29548	733 (2.5)	1.10 (0.98-1.24)	36846	3519 (9.6)	1.04 (0.97-1.11)
Q3	11187	199 (1.8)	1.04 (0.88-1.22)	30455	770 (2.5)	1.11 (0.98-1.25)	38631	3696 (9.6)	1.03 (0.96-1.11)
Q2	11109	163 (1.5)	0.96 (0.80-1.16)	31082	776 (2.5)	1.09 (0.96-1.25)	39495	4041 (10.2)	1.10 (1.02-1.19)
Q1 (Least deprived)	10052	223 (2.2)	1.17 (0.98-1.40)	31249	1096 (3.5)	1.50 (1.32-1.70)	42170	4889 (11.6)	1.25 (1.16-1.34)
Access to HPV vaccination[c]									
T1 (Low access)	16658	287 (1.7)	Ref.	44055	981 (2.2)	Ref.	55055	5128 (9.3)	Ref.
T2	21273	352 (1.7)	0.93 (0.81-1.06)	59044	1599 (2.7)	1.17 (1.07-1.28)	75578	7698 (10.2)	1.08 (1.02-1.14)
T3 (High access)	14928	297 (2)	0.94 (0.81-1.09)	42328	1263 (3)	1.17 (1.05-1.29)	54740	5862 (10.7)	1.09 (1.03-1.16)

Abbreviations: HPV, Human Papillomavirus; ADI, Area Deprivation Index

a The total sum of Target population and Cumulative HPV vaccination in ADI is less than that in the access indicator, because ADI cannot be calculated in areas with fewer than 50 households.

b Multivariable analysis was adjusted as follows: ADI, access to medical facilities providing HPV vaccination, generation of birth fiscal year, vaccination period.

c Number of facilities are as follows: T1, 0-5 facilities; T2, 6-10 facilities; T3, 11-26 facilities.

© 2025 Oka E et al. *JAMA Network Open.*

eTable 5. Cumulative Completed Dose HPV Vaccination Coverage by Neighborhood-Based Indicators in 2022

Exposure	Target population No.[a]	Cumulative HPV Vaccination No. (%)[a]	p-value[b]
**Routine + Catch-up vaccination**			
ADI			0.01
Q5 (Most deprived)	28078	1668 (5.9)	
Q4	36846	2390 (6.5)	
Q3	38631	2552 (6.6)	
Q2	39495	2757 (7.0)	
Q1 (Least deprived)	42170	3538 (8.4)	
Access to HPV vaccination[c]			0.11
T1 (Low access)	55055	3423 (6.2)	
T2	75578	5379 (7.1)	
T3 (High access)	54740	4105 (7.5)	
**Routine vaccination**			
ADI			0.01
Q5 (Most deprived)	28078	980 (3.5)	
Q4	36846	1417 (3.8)	
Q3	38631	1572 (4.1)	
Q2	39495	1666 (4.2)	
Q1 (Least deprived)	42170	2332 (5.5)	
Access to HPV vaccination[c]			0.11
T1 (Low access)	55055	2074 (3.8)	
T2	75578	3317 (4.4)	
T3 (High access)	54740	2577 (4.7)	
**Catch-up vaccination**			
ADI			0.05
Q5 (Most deprived)	28078	688 (2.5)	
Q4	36846	973 (2.6)	
Q3	38631	980 (2.5)	
Q2	39495	1091 (2.8)	
Q1 (Least deprived)	42170	1206 (2.9)	
Access to HPV vaccination[c]			0.11
T1 (Low access)	55055	1349 (2.5)	
T2	75578	2062 (2.7)	
T3 (High access)	54740	1528 (2.8)	

© 2025 Oka E et al. *JAMA Network Open.*

eTable 5. Cumulative Completed Dose HPV Vaccination Coverage by Neighborhood-Based Indicators in 2022 (continued)

Abbreviations: HPV, Human Papillomavirus; ADI, Area Deprivation Index

a The total sum of Target population and Cumulative HPV vaccination in ADI is less than that in the access indicator, because ADI cannot be calculated in areas with fewer than 50 households.

b The Jonckheere-Terpstra test was performed to identify trends in Cumulative HPV Vaccination Coverage.

c Number of facilities are as follows: T1, 0-5 facilities; T2, 6-10 facilities; T3, 11-26 facilities.

© 2025 Oka E et al. *JAMA Network Open*.

eTable 6. Association Between Neighborhood-Based Indicators and Cumulative Completed Dose HPV Vaccination Coverage in 2022

Exposure		Univariable analysis PR (95%CI)	Multivariable analysis[a] PR (95%CI)
**Routine + Catch-up vaccination**			
ADI			
	Q5 (Most deprived)	Ref.	Ref.
	Q4	1.09 (1.00-1.19)	1.07 (0.99-1.16)
	Q3	1.11 (1.02-1.21)	1.08 (0.99-1.17)
	Q2	1.18 (1.08-1.28)	1.14 (1.05-1.24)
	Q1 (Least deprived)	1.41 (1.30-1.54)	1.35 (1.24-1.46)
Access to HPV vaccination[b]			
	T1 (Low access)	Ref.	Ref.
	T2	1.14 (1.07-1.22)	1.11 (1.05-1.18)
	T3 (High access)	1.21 (1.12-1.30)	1.12 (1.05-1.20)
**Routine vaccination**			
ADI			
	Q5 (Most deprived)	Ref.	Ref.
	Q4	1.10 (0.98-1.24)	1.10 (0.99-1.22)
	Q3	1.17 (1.04-1.31)	1.14 (1.03-1.27)
	Q2	1.21 (1.08-1.35)	1.21 (1.09-1.35)
	Q1 (Least deprived)	1.58 (1.42-1.77)	1.51 (1.37-1.68)
Access to HPV vaccination[b]			
	T1 (Low access)	Ref.	Ref.
	T2	1.17 (1.07-1.27)	1.12 (1.04-1.21)
	T3 (High access)	1.25 (1.14-1.37)	1.14 (1.05-1.23)
**Catch-up vaccination**			
ADI			
	Q5 (Most deprived)	Ref.	Ref.
	Q4	1.08 (0.96-1.21)	1.04 (0.94-1.17)
	Q3	1.04 (0.92-1.16)	1.01 (0.90-1.13)
	Q2	1.13 (1.00-1.27)	1.08 (0.96-1.21)
	Q1 (Least deprived)	1.17 (1.05-1.30)	1.14 (1.02-1.27)
Access to HPV vaccination[b]			
	T1 (Low access)	Ref.	Ref.
	T2	1.11 (1.02-1.22)	1.10 (1.01-1.20)
	T3 (High access)	1.14 (1.04-1.25)	1.11 (1.01-1.22)

© 2025 Oka E et al. *JAMA Network Open.*

eTable 6. Association Between Neighborhood-Based Indicators and Cumulative Completed Dose HPV Vaccination Coverage in 2022 (continued)

Abbreviations: HPV, Human Papillomavirus; ADI, Area Deprivation Index; PR, Prevalence Ratio

a Multivariable analysis was adjusted as follows: ADI, access to medical facilities providing HPV vaccination, generation of birth fiscal year, vaccination period.

b Number of facilities are as follows: T1, 0-5 facilities; T2, 6-10 facilities; T3, 11-26 facilities.

© 2025 Oka E et al. *JAMA Network Open*.

eTable 7. Cross-Tabulation of Cumulative at Least 1-Dose HPV Vaccination Coverage in 2022 by ADI and Access

Exposure	Access to HPV vaccination[a]					
	T1 (Low access)		T2		T3 (High access)	
	Target population No.[b]	Cumulative HPV Vaccination No. (%)[b]	Target population No.[b]	Cumulative HPV Vaccination No. (%)[b]	Target population No.[b]	Cumulative HPV Vaccination No. (%)[b]
**Routine + Catch-up vaccination**						
ADI						
Q5 (Most deprived)	11443	1040 (9.1)	12928	1171 (9.1)	3707	328 (8.8)
Q4	12497	1091 (8.7)	16762	1675 (10)	7587	753 (9.9)
Q3	11435	1087 (9.5)	16336	1485 (9.1)	10860	1124 (10.3)
Q2	11016	1081 (9.8)	12715	1323 (10.4)	15764	1637 (10.4)
Q1 (Least deprived)	8637	829 (9.6)	16767	2043 (12.2)	16766	2017 (12)
**Routine vaccination**						
ADI						
Q5 (Most deprived)	11443	594 (5.2)	12928	623 (4.8)	3707	203 (5.5)
Q4	12497	609 (4.9)	16762	1000 (6)	7587	448 (5.9)
Q3	11435	659 (5.8)	16336	895 (5.5)	10860	703 (6.5)
Q2	11016	668 (6.1)	12715	824 (6.5)	15764	964 (6.1)
Q1 (Least deprived)	8637	539 (6.2)	16767	1367 (8.2)	16766	1350 (8.1)
**Catch-up vaccination**						
ADI						
Q5 (Most deprived)	11443	446 (3.9)	12928	548 (4.2)	3707	125 (3.4)
Q4	12497	482 (3.9)	16762	675 (4)	7587	305 (4)
Q3	11435	428 (3.7)	16336	590 (3.6)	10860	421 (3.9)
Q2	11016	413 (3.7)	12715	499 (3.9)	15764	673 (4.3)
Q1 (Least deprived)	8637	290 (3.4)	16767	676 (4)	16766	667 (4)

© 2025 Oka E et al. *JAMA Network Open.*

eTable 7. Cross-Tabulation of Cumulative at Least 1-Dose HPV Vaccination Coverage in 2022 by ADI and Access (continued)

Abbreviations: HPV, Human Papillomavirus; ADI, Area Deprivation Index

a Number of facilities are as follows: T1, 0-5 facilities; T2, 6-10 facilities; T3, 11-26 facilities.

b The total sum of Target population and Cumulative HPV vaccination in ADI is less than that in the access indicator, because ADI cannot be calculated in areas with fewer than 50 households.

© 2025 Oka E et al. *JAMA Network Open*.

eTable 8. Interaction Between ADI and Access[a]

Exposure	PR (95%CI)[b]
Q5 (Most deprived) x T1 (Low access)	Ref.
Q5 x T2	0.99 (0.89-1.12)
Q5 x T3 (High access)	0.96 (0.81-1.14)
Q4 x T1 (Low access)	0.96 (0.85-1.08)
Q4 x T2	1.08 (0.97-1.20)
Q4 x T3	1.07 (0.94-1.22)
Q3 x T1	1.03 (0.91-1.17)
Q3 x T2	0.98 (0.88-1.09)
Q3 x T3	1.11 (0.98-1.25)
Q2 x T1	1.06 (0.94-1.20)
Q2 x T2	1.13 (1.00-1.27)
Q2 x T3	1.12 (1.01-1.25)
Q1 (Least deprived) x T1	1.05 (0.91-1.21)
Q1 x T2	1.33 (1.18-1.49)
Q1 x T3	1.30 (1.16-1.46)

Abbreviations: HPV, Human Papillomavirus; ADI, Area Deprivation Index; PR, Prevalence Ratio

a. Number of facilities are as follows: T1, 0-5 facilities; T2, 6-10 facilities; T3, 11-26 facilities.

b Adjusted as follows: ADI, access to medical facilities providing HPV vaccination, generation of birth fiscal year, vaccination period.

© 2025 Oka E et al. *JAMA Network Open*.
