# Supplementary material for: Area Socioeconomic Status, Vaccination Access, and Female Human Papillomavirus Vaccination
Source: JAMA Netw Open. 2025 Mar 13;8(3):e250747. doi: 10.1001/jamanetworkopen.2025.0747 (PMC11907311; doi:10.1001/jamanetworkopen.2025.0747)
Supplement: Supplement 2. — Data Access Statement [file jamanetwopen-e250747-s002.pdf]

Data Sharing Statement

Oka. Area Socioeconomic Status, Vaccination Access, and Female Human Papillomavirus Vaccination. *JAMA Netw Open*. Published March 13, 2025.
doi:10.1001/jamanetworkopen.2025.0747

Data
Data available: No

Additional Information
Explanation for why data not available: Individual HPV vaccination data and tabulated data on the number of females vaccinated in Osaka City are not publicly available to maintain participant confidentiality, but are available from the corresponding author on reasonable request. Population data by one-year age group in Osaka City can be accessed at the following URL: https://www.city.osaka.lg.jp/contents/wdu290/opendata/. Tabulated data on the number of females vaccinated and population data for the whole of Japan can be accessed at the following URL: https://www.mhlw.go.jp/content/10601000/001092257.pdf, https://www.e-stat.go.jp/.
